# Genome-Wide Diversity and Phylogeography of *Mycobacterium avium* subsp. *paratuberculosis* in Canadian Dairy Cattle

**DOI:** 10.1371/journal.pone.0149017

**Published:** 2016-02-12

**Authors:** Christina Ahlstrom, Herman W. Barkema, Karen Stevenson, Ruth N. Zadoks, Roman Biek, Rowland Kao, Hannah Trewby, Deb Haupstein, David F. Kelton, Gilles Fecteau, Olivia Labrecque, Greg P. Keefe, Shawn L. B. McKenna, Kapil Tahlan, Jeroen De Buck

**Affiliations:** 1 University of Calgary, Calgary, Alberta, Canada; 2 Moredun Research Institute, Penicuik, Scotland; 3 University of Glasgow, Glasgow, Scotland; 4 SaskMilk, Regina, Saskatchewan, Canada; 5 University of Guelph, Guelph, Ontario, Canada; 6 Université de Montréal, Montréal, Québec, Canada; 7 Laboratoire d'épidémiosurveillance animale du Québec, Saint-Hyacinthe, Québec, Canada; 8 University of Prince Edward Island, Charlottetown, Prince Edward Island, Canada; 9 Memorial University of Newfoundland and Labrador, St. John’s, Newfoundland, Canada; University of Minnesota, UNITED STATES

## Abstract

*Mycobacterium avium* subsp. *paratuberculosis* (MAP) is the causative bacterium of Johne’s disease (JD) in ruminants. The control of JD in the dairy industry is challenging, but can be improved with a better understanding of the diversity and distribution of MAP subtypes. Previously established molecular typing techniques used to differentiate MAP have not been sufficiently discriminatory and/or reliable to accurately assess the population structure. In this study, the genetic diversity of 182 MAP isolates representing all Canadian provinces was compared to the known global diversity, using single nucleotide polymorphisms identified through whole genome sequencing. MAP isolates from Canada represented a subset of the known global diversity, as there were global isolates intermingled with Canadian isolates, as well as multiple global subtypes that were not found in Canada. One Type III and six “Bison type” isolates were found in Canada as well as one Type II subtype that represented 86% of all Canadian isolates. Rarefaction estimated larger subtype richness in Québec than in other Canadian provinces using a strict definition of MAP subtypes and lower subtype richness in the Atlantic region using a relaxed definition. Significant phylogeographic clustering was observed at the inter-provincial but not at the intra-provincial level, although most major clades were found in all provinces. The large number of shared subtypes among provinces suggests that cattle movement is a major driver of MAP transmission at the herd level, which is further supported by the lack of spatial clustering on an intra-provincial scale.

## Introduction

*Mycobacterium avium* subsp. *paratuberculosis* (MAP) is the causative bacterium of Johne’s disease (JD) in ruminants and is widespread in the global dairy industry [1]. A potential association between MAP and Crohn’s disease in humans is increasing pressure to reduce the prevalence in dairy herds by breaking the transmission cycle [2,3]. This can be achieved within herds by eliminating exposure of calves to infected feed and feces and between herds by preventing the introduction of MAP-infected animals [4]. Despite this knowledge, the herd-level prevalence of JD in Canada remains high [5].

MAP is a Gram-positive, acid-fast bacillus classified into two broad strain types–Type I and Type II [6]. Additional types have also been described, however Type III is now understood to be a sub-lineage of Type I [7] and “Bison type” (Type B) is a sub-lineage of Type II (Bryant et al., submitted for publication). Subtyping of isolates beyond the broad strain types has largely relied on repetitive regions of the genome; however, this limits the ability to infer true evolutionary relationships ([8]; Bryant et al., submitted for publication). The relatively high discriminatory ability of short sequence repeat (SSR) [9] and variable number tandem repeat (VNTR) typing [10] has motivated researchers to use these tools to investigate the diversity of MAP in several countries and host species (see [11] for a review). In Canada, the genetic structure of MAP was analyzed at a provincial level using a combination of SSR and VNTR loci [12]. Two genotypes accounted for 43% of the isolates and the majority of the remaining isolates differed by a single, potentially unstable, locus. Thus, more appropriate genotyping tools are required to capture the true population diversity necessary for molecular epidemiological analyses. Whole genome sequencing (WGS) provides a means to identify informative, stable molecular markers that can subsequently be used to assess the population structure in a broader scope [13].

A thorough understanding of the population structure of mycobacteria at multiple geographical scales can provide an important framework to investigate transmission in an epidemiological context as well as subtype-specific characteristics, such as virulence, transmissibility, and immunogenicity. WGS has been used to study the persistence and recent transmission events of *M*. *bovis* in cattle and badgers in UK dairy herds, improving the understanding of disease dynamics at small spatial scales [14]. Disease diversity has also been attributed to strain diversity in a number of pathogens, including *Mycobacterium tuberculosis* [15]. Strain-specific differences in virulence and pathogenicity of MAP, however, have mainly focused on the major strain types [16–19], in which significant differences were found between Type I and Type II isolates in their growth rates and intracellular survival.

An understanding of the true genetic diversity of MAP is needed to accurately assess subtype-specific phenotypes and virulence characteristics. Additionally, rational vaccine development and potentially effective management practices rely on a comprehensive assessment of circulating MAP subtypes and their distribution across different provinces. The objectives of this study were to A) determine the diversity and evolutionary relationship of MAP strain types in Canadian dairy farms from all 10 provinces using WGS, B) contextualize this diversity through comparison to a diverse global collection of MAP isolates, and C) assess the phylogeography of Canadian MAP isolates on a national and provincial level.

## Materials and Methods

### Canadian isolate selection and DNA preparation

Canadian MAP isolates were obtained from environmental and individual cow fecal samples collected from regional JD control initiatives in British Columbia, Alberta, Saskatchewan, Ontario, Québec, and the Atlantic region (New Brunswick, Nova Scotia, Prince Edward Island and Newfoundland), as detailed previously [5,8,20]. Additional MAP isolates were obtained from the Alberta Ministry of Agriculture and Rural Development (n = 22), representing individual cow fecal samples from different dairy herds in Alberta sampled between 1999 and 2002. Environmental manure samples from manure storage areas were also obtained from five dairy operations in the province of Manitoba. MAP DNA from environmental manure samples from three dairy herds in Newfoundland was also obtained. The isolates included in this study were obtained from a total of 170 dairy herds, all with permission from the owners, and are presented in S1 Table. Only one isolate per herd was selected, with the exception of 11 herds (located in Alberta or Saskatchewan) in which two or three isolates were included that previous WGS analyses identified as genetically distinct [8]. MAP was grown from environmental manure samples and individual cow fecal samples using the TREK ESP Culture System reagents (TREK Diagnostics, Cleveland, OH, USA) in all provinces, with the exception of Québec, where isolates were cultured with the BACTEC MGIT 960 ParaTB culture system (Becton, Dickinson and company, Franklin Lakes, NJ, USA). Liquid culture broth of IS900 PCR positive samples [5,21] was plated onto Middlebrook 7H11 agar supplemented with OADC and 2 mg/L mycobactin J and a single MAP colony was substreaked for subsequent regrowth and genomic DNA extraction, as detailed previously [22]. Multiplexed DNA libraries were prepared using the Nextera XT sample preparation kit (Illumina, San Diego, CA, USA) according to manufacturer’s instructions.

### Whole genome sequencing and single nucleotide polymorphism (SNP) detection

Paired-end WGS was performed on pools of 24 samples using the MiSeq platform (Illumina, San Diego, CA, USA) with a read length of either 250 or 300 base pairs. Reads were deposited in the sequence read archive (accession number SRP067719 and SRR060191). BWA [23] was used for reference mapping to the revised version of the MAP K10 genome (NCBI Sequence Read Archive study SRR060191) [24,25] and variant sites were identified using SAMtools and bcftools [26]. Variant sites within insertion elements and PE/PPE regions were discounted and subsequent filtering criteria [8] were used to minimize false-positives. Filtering criteria ensured at least 2 high quality SNPs on both the forward and reverse strands, a mapping quality >40, and a heterozygosity <5% to assign a position as either the “reference” or “alternate” allele. Isolates that failed the filtering criteria at a particular site was treated as an “unknown base” and assigned a value of “N”.

### Global sequence selection

MAP isolates (n = 135) from 22 countries were collected at Moredun Research Institute (MRI) having been selected in order to maximize the global genetic diversity. WGS and data analysis was performed as detailed elsewhere (Bryant et al., submitted for publication). A total of 26 sequences were selected from this collection for comparison to sequences from Canadian isolates based on their phylogenetic position and availability of metadata, such as year of isolation and host. All major phylogenetic clades were represented, including MAP types I, II, III, and B and raw sequencing reads were processed alongside the Canadian sequences, as described above. Additional sequences from the MRI global collection were subsequently analyzed using the same methods in a separate analysis to refine Type III and Type B isolates in the phylogeny.

### Phylogenetic analyses

Concatenated variant sites were used for all phylogenetic analyses. Maximum likelihood phylogenetic trees were created using PhyML [27] with the TPM1uf nucleotide substitution model, as determined by jModelTest (version 2.1.7) [28]. Node support was evaluated with 100 bootstrap pseudoreplications. Phylogenetic trees were visualized using FigTree (version 1.4.2), and iTOL [29] was used to annotate the tips with geographical location.

The phylogenetic signal was evaluated using the likelihood mapping function in TREE-PUZZLE [30,31]. This method determines the support for internal branches of a phylogeny and the phylogenetic content of a dataset by plotting 10,000 randomly selected quartets in a likelihood-mapping diagram. The percentage of quartets that cannot be reliably resolved demonstrates the strength of the phylogenetic signal. Additionally, the nucleotide alignment was tested for the presence of recombinant sequences using the pairwise homoplasy index (PHI) test [32] in SplitsTree (version 4.13.1) [33] with a significance threshold of p ≤ 0.05. A rarefaction analysis was performed to determine the subtype richness among different Canadian provinces. Subtypes, or clades, were assigned using a strict and relaxed definition; the strict definition divided the isolates into nine divergent subtypes based on a shared branch length of at least 50 SNPs and the relaxed definition, selected to capture the majority of subpopulations in the phylogeny, divided the isolates into 32 subtypes. The function RAREFY in the package VEGAN (version 2.2–1) was implemented in R, version 3.1.2 [34].

The phylogenetic association between MAP and geographical location was tested using BaTS (Bayesian Tip Significance Testing) [35]. This program tests the null hypothesis of no correlation between phylogeny and geographic location by performing randomization tests from a posterior set of trees generated through a Bayesian Markov chain Monte Carlo analysis. A posterior set of trees was generated using the general time-reversible (GTR) substitution model, a lognormal relaxed molecular clock, and a constant-sized coalescent model implemented in BEAST (version 1.8.0) [36]. BaTS takes into consideration phylogenetic uncertainty when testing the association index, parsimony score, and the monophyletic clade size statistic. The phylogenic association was tested with province of origin as well as with three regions (each between 43,000 and 45,000 square Km) within Alberta for which the location of farm origin was known (n = 58; Region 1, north of Red Deer; Region 2, between Red Deer and Calgary; Region 3, south of Calgary). A p-value ≤ 0.05 was regarded as statistically significant. Exact binomial tests were performed to test the underrepresentation of regions in particular phylogenetic clades using the exact binomial test implemented in R [34].

## Results

### Canadian and global isolates

**Fig 1:**
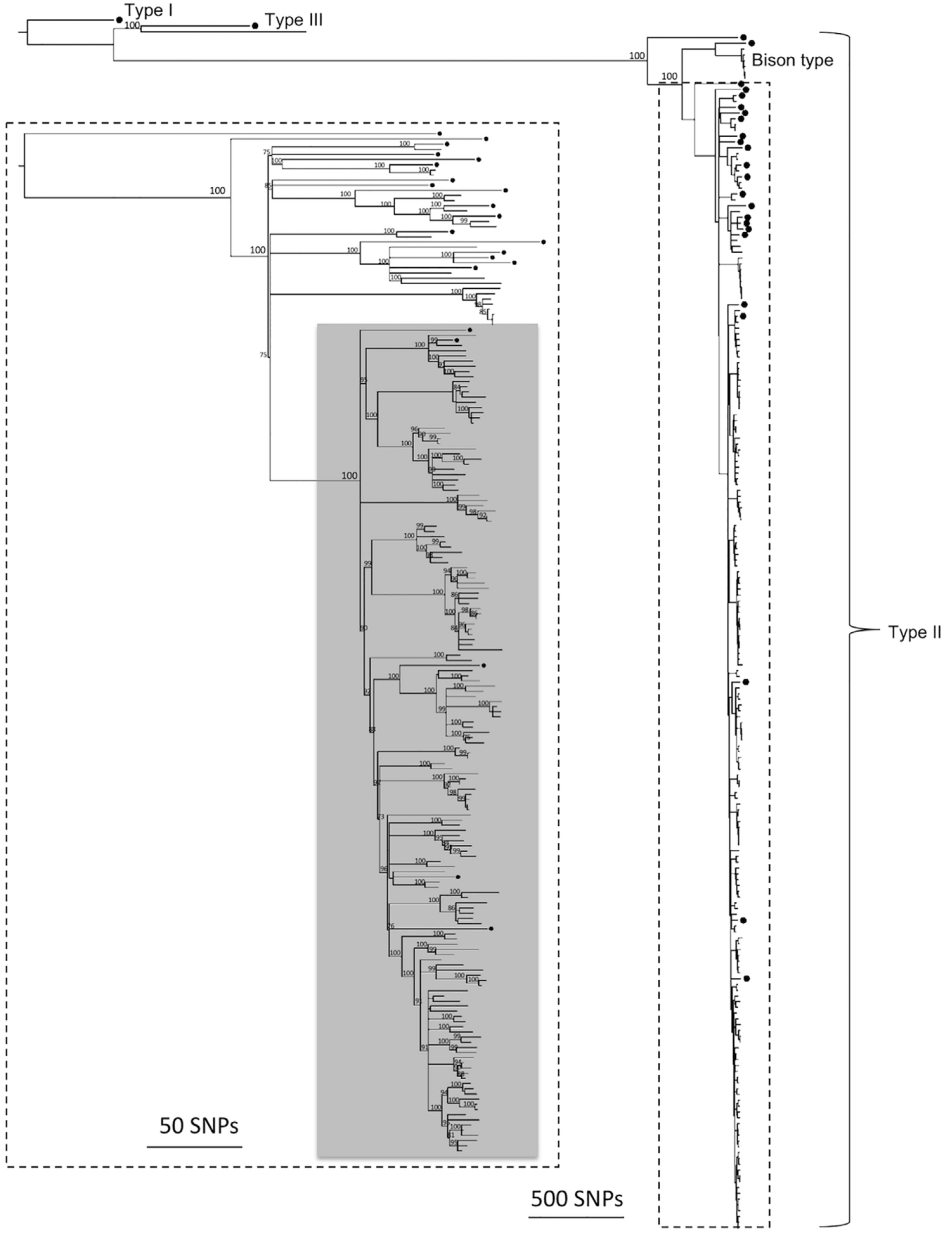
Maximum likelihood phylogenetic tree of sequenced MAP isolates. Phylogenetic tree of global (n = 26, labeled with a black dot (•)) and Canadian (n = 182) isolates based on 9,670 variant sites using the TPM1uf nucleotide substitution model. The tree is rooted to the Type I sequence. A magnification of the phylogeny excluding the Type I, III, and B isolates is outlined in dotted lines. The dominant subtype is shaded in grey. Bootstrap values with branch support ≥ 70% are displayed.

**Fig 2:**
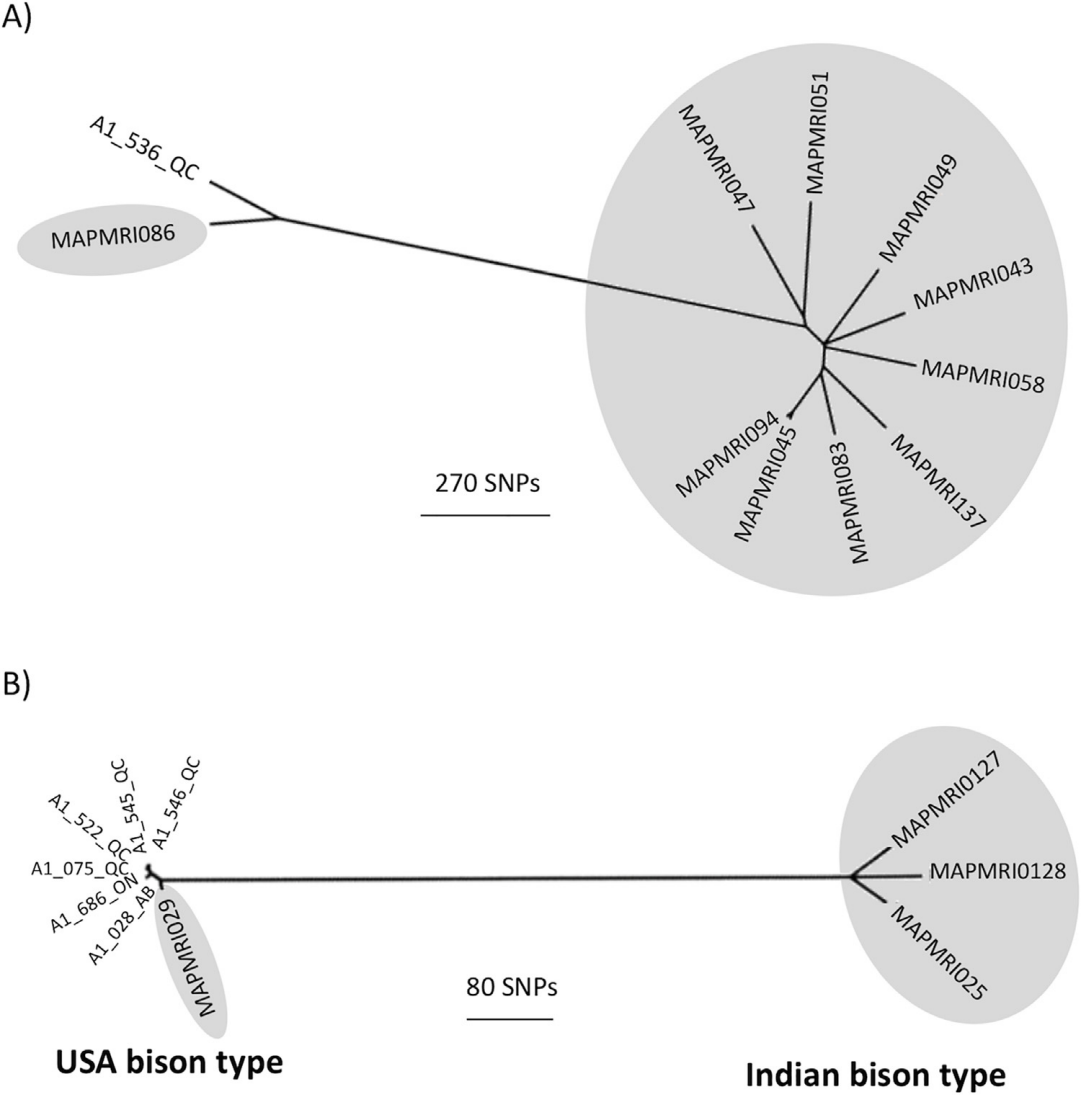
Maximum likelihood phylogenetic trees of Canadian and global MAP isolates. A) Phylogenetic tree of one Canadian and ten global Type III isolates based on 5,416 concatenated variant sites. B) Phylogenetic tree of six Canadian and four global “Bison type” isolates based on 403 concatenated variant sites. The global sequences are shaded in grey and branches are labeled according to the isolate ID.

A total of 9,670 variant sites were identified in 182 MAP isolates from Canada and 26 global isolates. The average depth of coverage of the Canadian and global isolates was 29 and 139, respectively; the average depth of coverage and number of raw reads for each isolate is presented in S1 Table. Results from the maximum likelihood phylogenetic analysis of concatenated variant sites are presented in Fig 1. One isolate from Québec (A1_536_QC) was most closely related to a Type III isolate from the global collection (MAPMRI051), with 1,411 pairwise SNP differences. The Canadian Type III sequence was subsequently compared to an additional nine Type III sequences from the global collection, which identified a deer isolate from the Czech Republic (A1_536_QC) as the closest relative (313 pairwise SNP differences) (Fig 2A).

The remaining 181 Canadian isolates clustered with the Type II global isolates. A dominant subtype included 155 of the Canadian and five of the global isolates. Additionally, six Canadian isolates from three provinces (Alberta, Ontario, and Québec) were most closely related to the Type B isolate from the global collection (MAPMRI0128). To further investigate the relationship between all Type B isolates, an additional three global Type B sequences (from isolates originating from India, the Czech Republic, and the USA) were analyzed with the six Canadian sequences. A total of 403 SNPs were identified among Type B sequences and the phylogenetic analysis based on these SNPs revealed close similarity between the Canadian isolates and an isolate originating from a bison herd in the USA (MAPMRI029) (Fig 2B). The USA and Canadian sequences had between eight and 31 pairwise SNP differences, with the USA sequence most closely related to the sequence from Alberta (A1_028_AB).

### Canadian isolates

6,604 variant sites were identified in 182 Canadian isolates, although nearly half (3,022) were unique to the Type III isolate. 3,582 SNPs were identified within the Type II isolates, including the six Type B isolates. Likelihood mapping analysis indicated a strong phylogenetic signal, with greater than 80% of the quartets fully resolved (S1 Fig). The PHI test did not indicate statistically significant evidence for recombination, with p = 0.73.

A total of nine divergent MAP subtypes were found in Canada, of which eight were classified as Type II. Within the Type II isolates, 86% belonged to the dominant subtype (subtype H), with the remaining subtypes each representing less than 4% of the population (subtype A, B, C, D, E, F, and G represented 3, 0.5, 1, 3, 2, 4, 0.5% respectively). Two of those subtypes (B and G) were each represented by a single isolate. Subtypes that contained more than one isolate included as few as one region or as many as all seven. Within the dominant subtype in particular, long internal branches differentiated this subtype into several subpopulations that were differentiated in the rarefaction analyses using the relaxed subtype definition.

**Fig 3:**
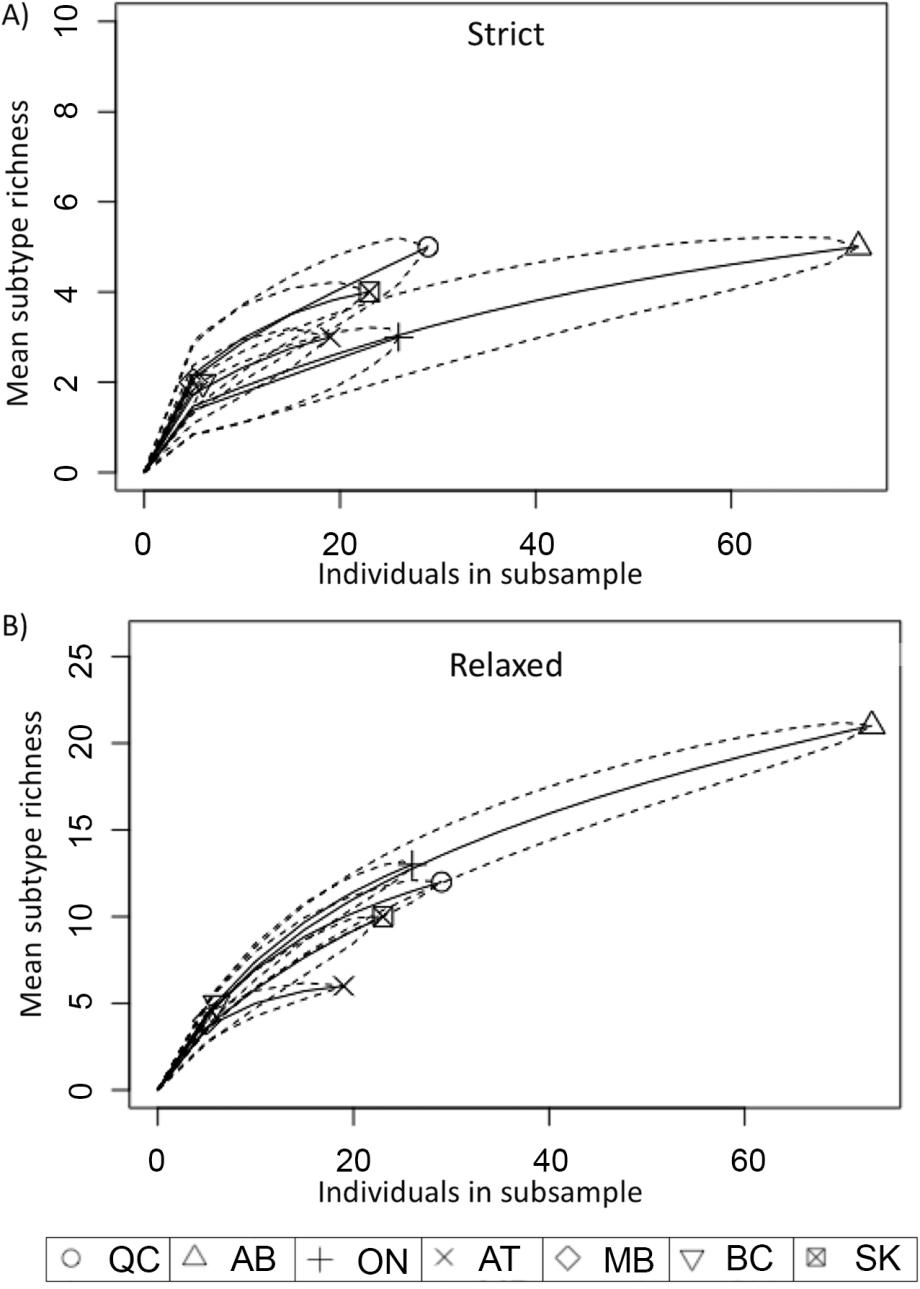
Rarefaction curves indicating the mean subtype richness of each region at different sampling efforts. Rarefaction curves were generated using the function rarefy in the R package Vegan using A) a strict subtype definition (9 total subtypes) and B) a relaxed subtype definition (32 total subtypes). 95% confidence intervals are indicated by a dotted line. The 7 regions are labeled with shapes indicated in the key at the bottom of the figure. Regions are abbreviated as follows: Québec = QC, Alberta = AB, Ontario = ON, the Atlantic region = AT, Manitoba = MB, British Columbia = BC, and Saskatchewan = SK.

The rarefaction analyses quantified the subtype richness at two different cutoffs to capture the diversity based on more recent and more ancestral SNPs given a range of sampling efforts for each region (Fig 3). Using the strict, more ancestral subtype definition Québec displayed more diversity than Alberta at a sampling effort of 25 samples. However, at smaller sample sizes the 95% confidence intervals of all provinces overlapped, limiting the ability to interpret levels of diversity. The rarefaction analysis based on 32 subtypes only identified a difference in subtype richness in the Atlantic region (Fig 3B), where a lower level of diversity was observed.

**Fig 4:**
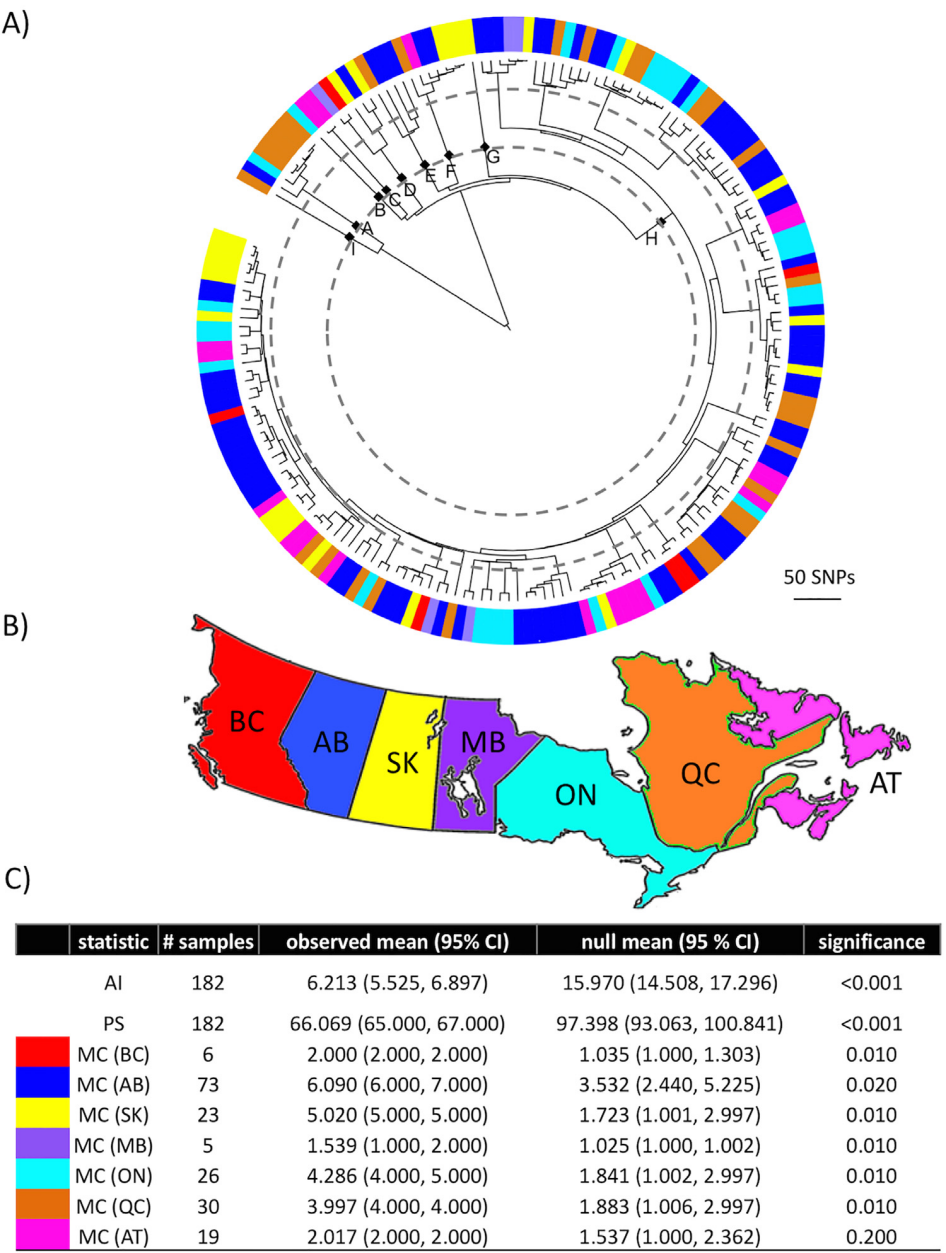
Phylogenetic clustering of MAP isolates among Canadian regions. A) Circularized maximum likelihood phylogenetic tree of 182 Canadian MAP isolates, rooted to the Type III isolate. Tips are colored according to province of origin (Alberta = blue, Ontario = light blue, British Colombia = red, Québec = orange, Saskatchewan = yellow, the Atlantic provinces = pink, Manitoba = purple). The branches leading to the nine subtypes are labeled with a square. Dotted lines indicate the threshold for the different subtype definitions used in the rarefaction analysis. B) Map of the 7 Canadian regions in which MAP isolates were obtained. C) The statistics (AI = association index, PS = parsimony score, MC = monophyletic clade size statistic), number of samples from each of the 7 regions, observed mean, null mean, and significance (p-value) are presented. Low AI and PS scores indicate a strong association, whereas high MC scores indicate a strong association.

**Fig 5:**
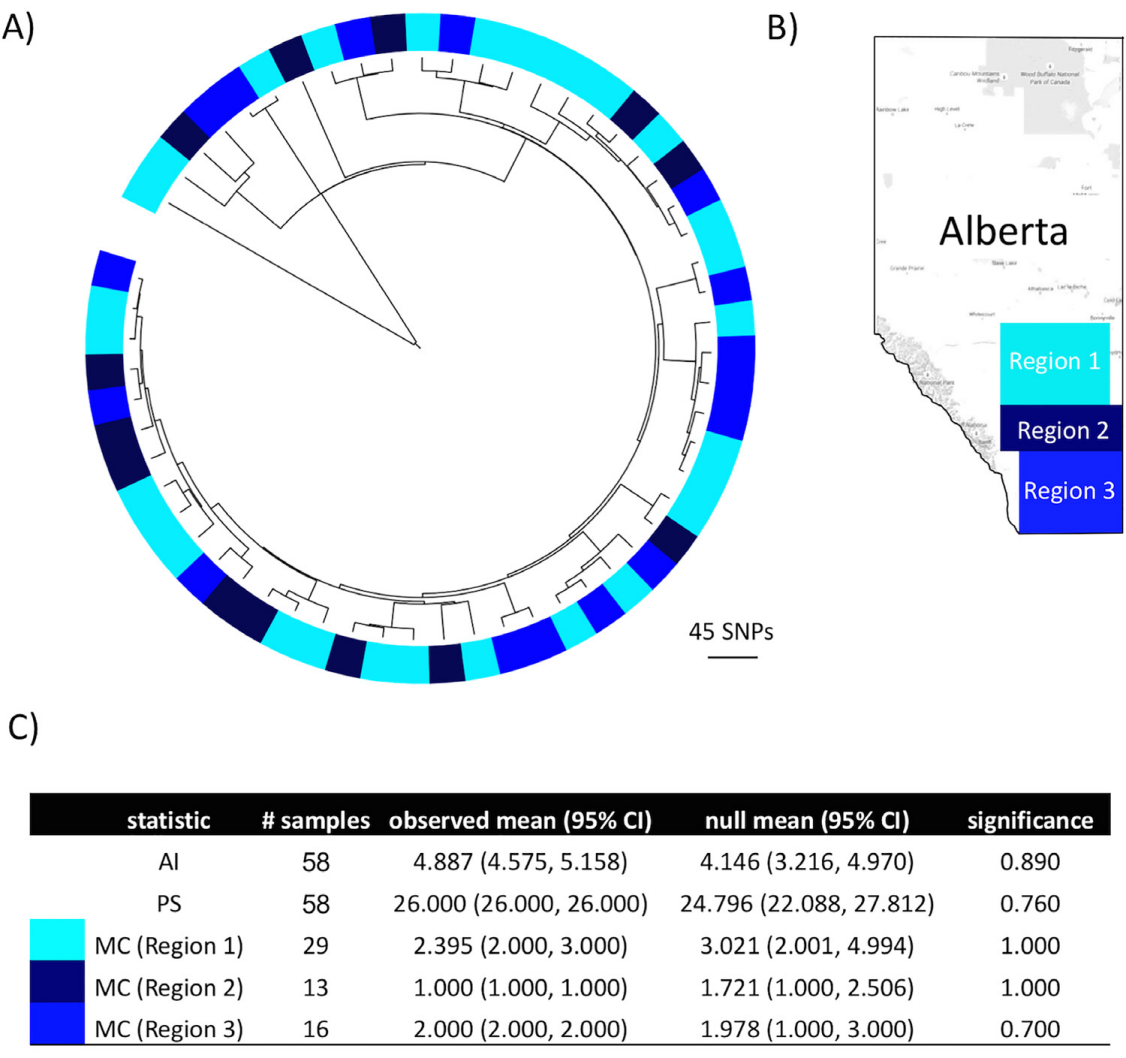
Phylogenetic clustering of MAP isolates in three regions in Alberta. A) Circularized maximum likelihood phylogenetic tree of 58 Alberta MAP isolates. Tips are colored according to the geographical location of originating farms (Region 1 = light blue, Region 2 = dark blue, Region 3 = blue). B) Map of Alberta with the three regions indicated by their respective colors. C) BaTS analysis of the phylogeny-region association. The statistics (AI = association index, PS = parsimony score, MC = monophyletic clade size statistic), number of samples from each region, observed mean, null mean, and significance (p-value) are presented. Low AI and PS scores indicate a strong association, whereas high MC scores indicate a strong association.

The presence of phylogeographic structure was tested using the program BaTS. When sequences were labeled according to province of origin, the parsimony score (PS) and association index (AI) statistics strongly rejected the null hypothesis of panmixis (Fig 4C). The monophyletic clade size statistic (MC) quantified the strength of each phylogeny-location association. With the exception of the Atlantic region, all regions demonstrated a significant association (p < 0.05). The MC statistics from British Columbia and Manitoba should be interpreted with caution due to their small sample size. A phylogenetic tree with the tips colored according to the province in which the isolate was sampled is depicted in Fig 4A. There were noticeably missing regions in subpopulations within the dominant clade. Isolates from the Atlantic region were not represented in the basal portion of the dominant clade (H) and isolates from Québec were not present in the distal portion of the dominant clade. An exact binomial test indicated these two regions were significantly underrepresented in those clades, with p = 0.018 and p = 0.005, respectively. When Alberta isolates were tested according to their location within the province, significant associations were not observed, as PS, AI, and all three MC statistics resulted in p > 0.05 (Fig 5).

## Discussion

The WGS comparison of 182 Canadian MAP isolates originating from dairy herds in all 10 provinces to 26 diverse global isolates revealed the evolutionary relationship and subtype diversity of MAP. Although the proportion of subtypes could not be directly compared between the two datasets (because the global collection was selected to maximize genetic diversity), there were subtypes in the global collection that were not found in Canada as well as global isolates that were interspersed with the Canadian isolates. This indicates that out of the known global diversity only a subset of subtypes has been introduced into Canada.

To our knowledge, this is the first report of Type III MAP isolated from a Canadian dairy farm. Furthermore, the identification of Type B isolates from three Canadian provinces, obtained through routine monitoring as part of JD control programs rather than through targeting of potential Type B sources, indicates that this strain type is more common than previously understood [12]. The close genetic relatedness of the USA Type B isolate [37] to the Canadian Type B isolates suggests a common ancestor within the past 25 years, given an estimated substitution rate of 0.3 SNPs per genome per year (Bryant et al., submitted for publication).

The dominant subtype included 86% of all Canadian MAP isolates. Multiple introduction events of this MAP subtype from other countries is likely, as five global isolates fell within the dominant clade and were often basal to the Canadian isolates. The large number of subpopulations within this type also supports this hypothesis. The current population structure of MAP in Canada was likely shaped by the importation of cattle from a limited number of countries (although uneven sampling makes it impossible to definitively identify the direction of transmission) and the spread of certain successful subtypes in the Canadian dairy environment. To further support the hypothesis that some subtypes may have a selective advantage, both *in vivo* and *in vitro* investigations into phenotypic differences, such as growth rates and invasion efficiencies, is warranted. Future vaccine development studies and molecular epidemiological analyses should also consider the relative frequencies of MAP subtypes in Canada with a focus on the dominant subtype.

The province-level diversity was assessed through rarefaction analyses. The greater diversity of divergent subtypes (using the strict definition) in Québec, which suggests a larger bacterial effective population size in this province, may be a reflection of their large number of dairy herds. In 2014, 5,894 dairy herds were operating in Québec, compared to just 566 in Alberta [38]. However, Saskatchewan, with only 166 dairy herds, exhibited greater MAP diversity than Ontario, with 3,926 herds, which doesn’t fit the hypothesis that larger host population size and larger bacterial population size drive bacterial diversity. The rarefaction analysis with a more relaxed subtype definition indicated there is not a substantial difference in subtype diversity between provinces, with the exception of lower diversity seen in the Atlantic region. This complex distribution of MAP subtypes across the country has likely been shaped by herd management behaviors (such as biosecurity measures, JD testing strategy, cow-calf manure contact, colostrum management, etc.) that may select for certain MAP subtypes as well as the expansion of the Canadian dairy industry over the past two centuries, which may have contributed to repeated introductions.

The phylogeography was assessed at the between and within province level. The MC statistic was not significant in the Atlantic region, which includes four provinces separated by major geographical barriers. The grouping into one region may not be appropriate even though all four provinces were managed by the same JD control program [39]. There were large subpopulations within the dominant clade that did not include isolates from certain regions. This observation supports the rejection of the null hypothesis of panmixis at the between province level, even though there was still considerable overlap of subtypes across the country. There are several elements that could lead to an absence/low occurrence of subtypes in particular regions, such as local environmental factors, regional disease control strategies, a lack of introduction, or incomplete sampling. Animal trade is the probable driver of the shared subtypes between provinces. The movement of dairy cattle between herds in Canada is extensive but not well documented, as producers are not required to report livestock movements. A network analysis of dairy cattle movements in Ontario between 2004 and 2006 estimated approximately 15% of dairy cattle shipments left the province (the majority going to Québec) and 10–20% remaining within the same postal code [40].

At the within-province level, MAP isolates from three regions in Alberta were not significantly correlated with the phylogeny. This is in contrast to *Mycobacterium bovis* in UK cattle, where geographic clustering of types at a smaller geographic scale is observed, and where wildlife populations are implicated as a potential reservoir for local and regional transmission [14,41]. In Alberta, however, the lack of spatial association suggests spatially-localized transmission mechanisms, such as wildlife, have not been a major driver of MAP transmission. Spatiotemporal clustering of one common MAP genotype was previously observed in Québec dairy herds [12], but genotypes were defined based on repetitive loci and may not have been a reliable measure of relatedness [8]. An understanding of dairy cattle movements within and between all provinces will help clarify the phylogeography of MAP in Canada, as the movement to and from major livestock markets may be influential in disseminating MAP. Modeling cattle contact networks may allow prediction of the patterns of herd-to-herd MAP transmission, similar to the approach used to estimate the main drivers of *M*. *bovis* transmission in British cattle [42] or human tuberculosis transmission in British Columbia, Canada [43].

The disparity in the number of MAP isolates sequenced from each province likely influenced the BaTS phylogeny-trait association. Manitoba and British Columbia had very small sample sizes, so the presence of just two neighboring tips in the phylogeny produced a significant signal. Additionally, while province is an important variable in the movement of livestock, there may be regional clusters that were not investigated in this study. For example, dairy farms in western Ontario may be more likely to buy/sell animals to dairy farms in Manitoba than eastern Ontario. This emphasizes the need for additional data regarding livestock movements in Canada. The number of shared subtypes with the USA is also an important research question; over the past 10 years, Canada has imported more than 200,000 dairy cattle from the United States [44]. This highlights the importance of assessing the population structure of MAP in the United States using WGS to appropriately evaluate the contribution of international MAP transmission.

## Conclusions

Nine divergent MAP subtypes, including Type III and Type B, were identified in Canadian dairy herds, as well as a dominant subtype that comprised 86% of the Canadian isolates. The close similarity between the Type B isolates from the USA and Canada suggests a recent transmission event between bison and dairy cattle. The phylogenetic association of MAP isolates from different provinces indicates a degree of strain sharing within provinces, as well as interprovincial movement of MAP subtypes. Further analysis of the population structure with a larger set of isolates would potentially identify which factors influence the degree of subtype diversity between different regions in Canada. SNPs alone are not sufficient to track MAP transmission given its slow growth rate and the extensive, undocumented interprovincial cattle movement. However, integrating genetic data with animal movement data will help clarify MAP transmission dynamics at a regional and national scale.

## Supporting Information

S1 Fig Likelihood mapping analysis of 182 Canadian MAP isolates. The phylogenetic signal of the dataset was tested by plotting 10,000 quartets, in which the unresolved quartets (10.8%) are shown in the central region of the triangle. 80.3% of the quartets were fully resolved and 8.8% were partially resolved.
(TIFF)

S1 Table ID, origin, depth of coverage, and number of reads for each MAP isolate included in the study. (XLSX)
