# Supplementary figures and images for: Genome-Wide Diversity and Phylogeography of Mycobacterium avium subsp. paratuberculosis in Canadian Dairy Cattle
Source: PLoS One. 2016 Feb 12;11(2):e0149017. doi: 10.1371/journal.pone.0149017 (PMC4752300; doi:10.1371/journal.pone.0149017)

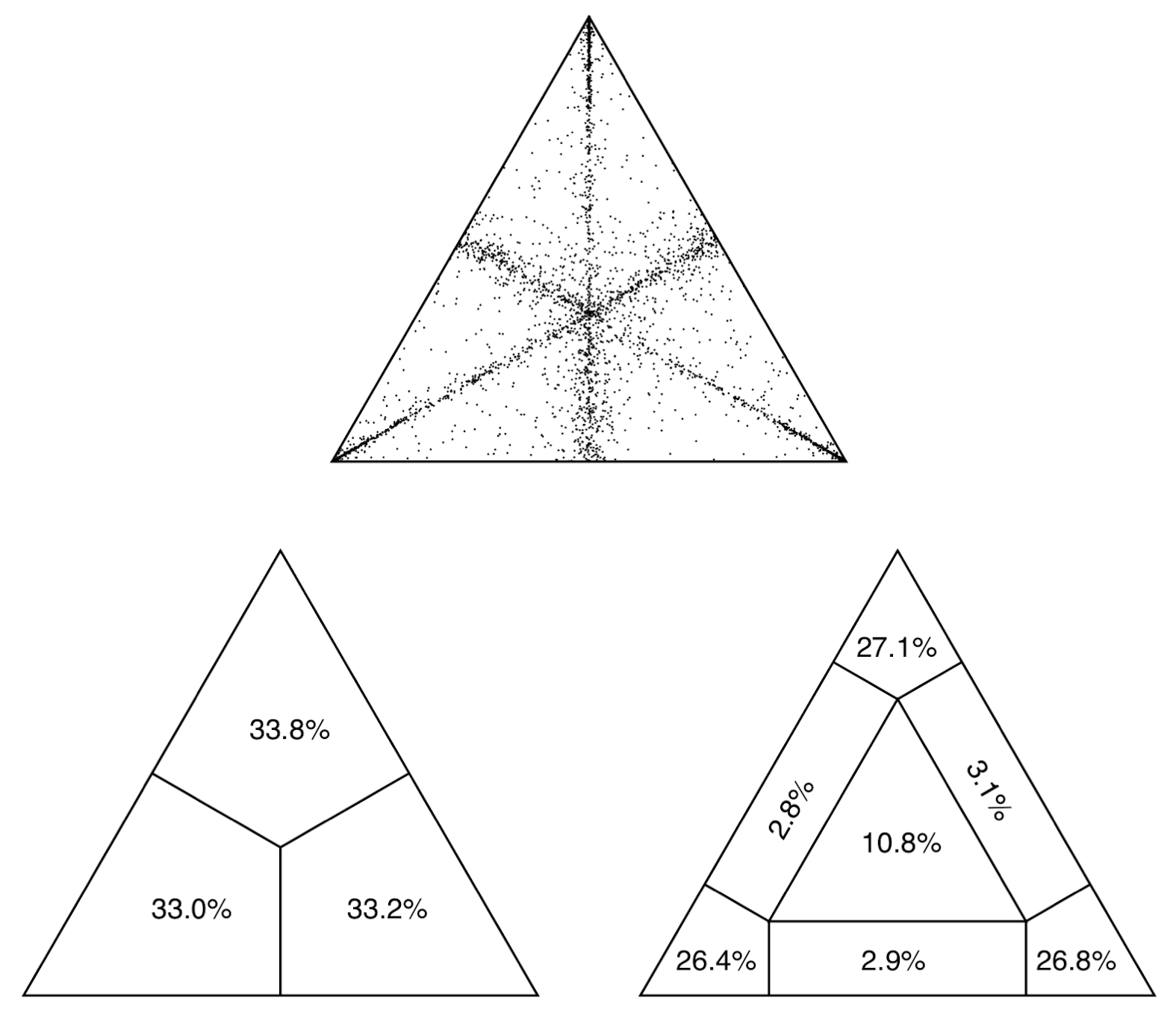

Supplement: S1 Fig — The phylogenetic signal of the dataset was tested by plotting 10,000 quartets, in which the unresolved quartets (10.8%) are shown in the central region of the triangle. 80.3% of the quartets were fully resolved and 8.8% were partially resolved. (TIFF) [file pone.0149017.s001.tiff]
